# Efficacy of* Brucea javanica* Oil Emulsion Injection Combined with the Chemotherapy for Treating Gastric Cancer: A Systematic Review and Meta-Analysis

**DOI:** 10.1155/2018/6350782

**Published:** 2018-05-02

**Authors:** Jia-Rui Wu, Shu-Yu Liu, Jia-Lian Zhu, Dan Zhang, Kai-Huan Wang

**Affiliations:** Department of Clinical Chinese Pharmacy, School of Chinese Materia Medica, Beijing University of Chinese Medicine, Beijing 100102, China

## Abstract

**Objective:**

This meta-analysis sought to assess the efficacy and safety of* Brucea javanica* oil emulsion injection (BJOEI) combined with chemotherapy for treating gastric cancer (GC).

**Method:**

Randomized controlled trials (RCTs) regarding BJOEI to treat GC were searched in PubMed, the Cochrane Library, Embase, the China National Knowledge Infrastructure Database (CNKI), the Wan-Fang Database, China Science and Technology Journal Database (VIP), and the Chinese Biomedical Literature Database (SinoMed) up to January 9, 2017. The clinical total effective rate, performance status, adverse drug reactions (ADRs), and other outcomes were analyzed with Review Manager 5.3 and Stata12.0 software.

**Results:**

13 RCTs involving 912 patients were included in the present meta-analysis. The results demonstrated that, compared with receiving chemotherapy alone, BJOEI combined with chemotherapy was more effective in improving clinical total effective rate (RR = 1.38, 95% CI: 1.22~1.56, *P* < 0.00001), performance status (RR = 1.63, 95% CI: 1.30~2.04, *P* < 0.00001), and relieving ADRs such as myelosuppression, neutropenia, thrombopenia, and liver damage. Statistically significant difference was observed between the experimental group and control group.

**Conclusion:**

The pooled analysis showed that using BJOEI on the basis of the chemotherapy had a remarkable therapeutic effect for patients with GC, whereas more evidence-based medical researches were required to further support our study.

## 1. Introduction

Gastric cancer is the fifth most common cancer and the second leading cause of cancer mortality worldwide [1]. More than 50% of patients undergo surgery, but even after a curative resection, about 60% of patients relapse locally or with distant metastases [2]. Traditional Chinese Medicine (TCM) holds that the occurrence and development of GC are mainly related to two factors: one is the loss of positive Qi; the other is the invasion of carcinogenic factors [3, 4]. By means of enhancing or inhibiting the immune response of patients with cancer, TCM modulates the immune ability to achieve the inhibition effect of tumor growth and metastases [5]. Served as an important part of the comprehensive treatment for GC, TCM is not only capable of reducing side effects of chemotherapy, but also able to alleviate the clinical symptoms of patients with advanced GC [6]. Comprised of oleum fructus bruceae, fabaceous lecithin, and glycerol, BJOEI takes petroleum ether extracts as raw material and purified soybean lecithin as emulsifier and is employed as adjunctive therapy in the treatment of lung carcinoma, brain metastasis of lung carcinoma, and gastrointestinal tumorigenesis [7–12]. On the basis of comprehensively collecting published RCTs, this research took advantage of meta-analysis to objectively evaluate the efficacy and safety of BJOEI for treating GC and also provided references for clinical decision-making.

## 2. Methods

### 2.1. Inclusion and Exclusion Criteria

RCTs regarding BJOEI combined with the chemotherapy for the treatment of GC could be involved in our study, whether using blinding methods or not. The inclusion criteria were as follows:

*Patients. *(1) The patients were confirmed by biopsy and postoperative pathological examination; (2) the patients had the measurable or evaluable lesions; (3) expected survival period was more than 2 months; (4) they received combined or single chemotherapy over 1 cycle; (5) there should be more than 1 month since the last treatment; (6) they should be without obvious chemotherapy contraindications and liver and kidney dysfunction. And there was no limitation of age, gender, race, or course and severity of disease.

*Interventions. *The normal dosage of BJOEI is 10–30 ml (which was diluted with 250 ml sterilized normal saline), once per day through an intravenous drip. Control group adopted chemotherapy alone, which is comprised of Oxaliplatin + Capecitabine (XELOX), paclitaxel (PTX) + Capecitabine (ECX), Tegafur Gimeracil Oteracil Potassium Capsule (S1) + Cisplatin (DDP), UFT + Folic acid (BC), Docetaxel + Capecitabine (DX), Oxaliplatin + calcium folinate + fluorouracil (FOLFOX4), Oxaliplatin (OXA) + Cisplatin + fluorouracil (CF) + Tegafur, MC/CF. The BJOEI group referred to BJOEI combined with the same chemotherapeutic drugs as control group.

*Outcomes. *The primary outcomes of this research were clinical total effective rate, performance status, and ADRs. The criterion of the curative effect met the WHO for solid tumors [26]. The clinical total effective rate was calculated by the following formula: the clinical total effective rate = [number of complete response (CR) patients + partial response (PR) patients]/total number of patients × 100%. Karnofsky performance score (KPS) was used to assess performance status, which increased above 10 points after treatment was considered as significant improvement.

RCTs which met the following criteria were excluded in this meta-analysis: (1) the method of generating random sequences that was wrong; (2) RCTs that are not available for the full text; (3) RCTs that did not meet the requirements of the intervention; (4) the evaluation standards that were not definite.

### 2.2. Literature Search

RCTs involving BJOEI combined with chemotherapy to treat GC were systematically retrieved by searching the following databases from inception to January 9, 2017: PubMed, the Cochrane Library, Embase, CNKI, the Wan-Fang Database, VIP, and SinoMed. The combination of MeSH terms and text words was applied into the retrieval. “Stomach Neoplasms” was regarded as the MeSH term. All the strategies were adapted by different databases. The strategies of PubMed were listed as follows:

  #1 “Stomach Neoplasms” [Mesh]
  #2 “Stomach Neoplasms^*∗*^” [Title/Abstract] OR “Gastric Cancer^*∗*^” [Title/Abstract] OR “Gastric Carcinoma” [Title/Abstract] OR “Gastric Neoplasm^*∗*^” [Title/Abstract] OR “Cancer of Stomach” [Title/Abstract] OR “Stomach Cancer^*∗*^” [Title/Abstract]
  #3 #1 OR #2
  #4 “Javanica oil emulsion injection” [Title/Abstract] OR “Yadanzi” [Title/Abstract] OR “Brucea javanica oil emulsion” [Title/Abstract] OR “Brucea javanica” [Title/Abstract]
  #5 #3 AND #4

### 2.3. Data Extraction and Quality Assessment

All data was extracted independently by two investigators (JW and SL), and any discrepancies between the reviewers were resolved by intercessor until consensus was reached. Data retrieved from the publications included author's name, the year of publication, the number of patients, average age, gender, the details about dosages and course of treatment, and the data of outcomes.

The quality assessment was conducted by the Cochrane risk of bias tool, which included random sequence generation, allocation concealment, blinding of participants and personnel, blinding of outcome assessment, incomplete outcome data, selective outcome reporting, and other sources of bias. Each item was classified into 3 ranks: “high,” “unclear,” or “low.”

### 2.4. Statistical Analysis

All the meta-analysis data utilized Review Manager 5.3 (Cochrane Collaboration, Oxford, UK) for synthesis and analysis. For outcomes, this meta-analysis chose relative risk (RR) to evaluate dichotomous outcomes, while using mean difference (MD) to assess continuous variables, each outcome numerical value was presented with 95% confidence intervals (95% CIs) as well. Heterogeneity between RCTs was analyzed by chi-square test and estimated by *I*^2^. Results of *P* ≥ 0.1 and *I*^2^ ≤ 50% suggested a lack of significant heterogeneity, and fixed-effect model was used accordingly, otherwise the random effect model conversely [27]. Publication bias was detected by Egger's test, and *P* < 0.05 was considered significant publication bias [28]. In addition, the sensitivity analysis was conducted to test the stability of results by Stata12.0 software.

## 3. Results

### 3.1. Literature Search and the Characteristics of Included RCTs

**Figure 1:**
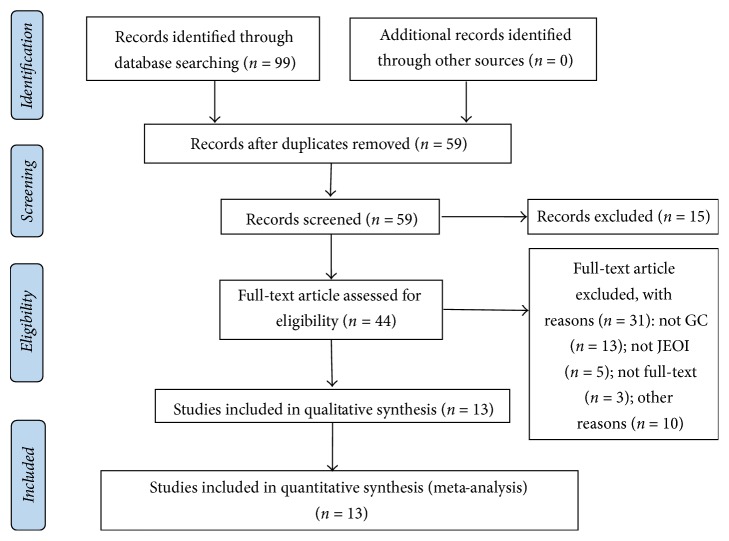
Meta-analysis profile summarizing trail flow.

A total of 99 articles (*n* = 912) were identified via a primary search of the aforementioned literature databases, from which 55 were excluded after titles and abstracts screening. Of the 44 RCTs remaining for full-text screening, we excluded case reports, animal experiments, editorials, letters, and reviews. And for duplicated RCTs, only the most updated and comprehensive ones were chosen. Ultimately, 13 related RCTs were identified. The detailed steps of literature search flow and screening process were depicted in Figure 1.

There were 467 and 445 patients in the BJOEI groups and chemotherapy group, respectively. Age span is from 24 to 85. Besides, the range of sample size was from 43 to 130 in included RCTs. Among all of RCTs male patients accounted for about 70%. What is more, there were 11 RCTs that adopted 30 ml/d in our research, 2 RCTs employed 20 ml/d, and the course of treatment was at least 14 days. Due to the diverse dosages of BJOEI, subgroup analysis may be taken into account. More details regarding these RCTs were presented in Table 1.

### 3.2. Quality Assessment

**Figure 2:**
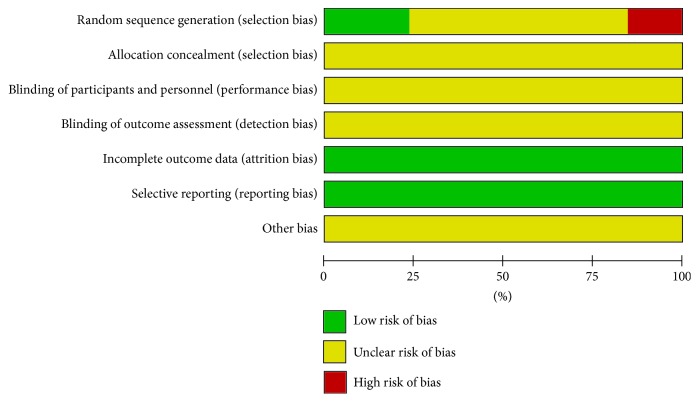
Risk of bias summary.

This meta-analysis utilized Review Manager 5.3 software to perform quality assessment. Only 2 RCTs [13, 25] adopted a random number table, and 1 RCT [21] used simple method of randomization. Therefore, selection bias was evaluated as “low risk.” 2 RCTs [17, 23] were divided into two groups in congruent with the hospitalized time, so the selection bias was remarked as “high risk.” And the other 8 RCTs [14–16, 18–20, 22, 24] did not illustrate how to implement randomized method; then the selection bias was labelled as “unclear risk.” Because none of the included RCTs reported concealment allocation, then selection bias due to allocation concealment, performance bias, and detection bias was deemed as “unclear risk.” Moreover, there was no case deficiency or selective reporting among included RCTs; hence, the attrition bias and reporting bias were assessed as “low risk.” Regarding other bias, 13 RCTs did not offer any details contributing to high risk, so this item was evaluated as “unclear risk.” Graphical description about quality assessment was shown in Figure 2.

### 3.3. Outcomes

#### 3.3.1. Clinical Total Effective Rate

**Figure 3:**
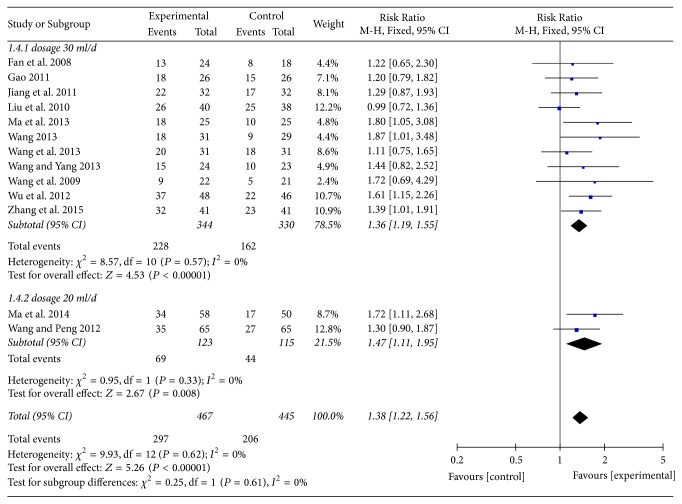
Meta-analysis in clinical total effective rate.

13 RCTs were available for clinical total effective rate. To explore potential effect differences in regard to clinical total effective rate, subgroup analysis was undertaken according to the different dosages of BJOEI among included RCTs, namely, dosage 30 ml/d and dosage 20 ml/d. As shown in Figure 3, the results indicated that there were statistically significant difference in clinical total effective rate between BJOEI intervention and control group in patients with 30 ml/d (RR = 1.36, 95% CI: 1.19~1.55, *Z* = 4.53, *P* < 0.00001) and 20 ml/d (RR = 1.47, 95% CI: 1.11~1.95, *Z* = 2.67, *P* = 0.008). The test for subgroup difference by dosages of BJOEI implied no significant difference between subgroups (*I*^2^ = 0%). Consequently, we were able to draw a conclusion that, compared with chemotherapy single, BJOEI combined with chemotherapy achieved superior effects for improving clinical effective rate of patients with GC.

#### 3.3.2. Performance Status

**Figure 4:**
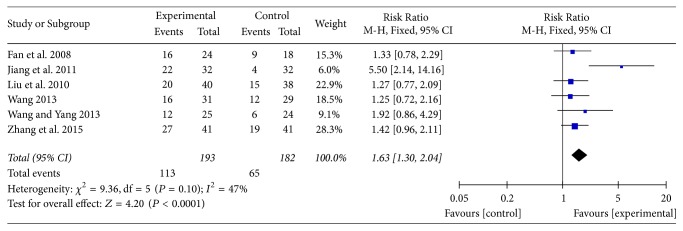
The forest plot of the performance status.

In total, 6 RCTs [14, 16, 17, 20, 22, 26] recorded the data of performance status of two groups and pooled results showed a small heterogeneity (*P* = 0.10, *I*^2^ = 47% <50%); therefore, the fixed-effect model was applied. Meta-analysis results demonstrated that BJOEI group experienced about 63% superiority in terms of this outcome compared with control group which only received chemotherapy, and the difference had statistical significance (RR = 1.63, 95% CI: 1.30~2.04, *Z* = 4.20, *P* < 0.00001; Figure 4).

#### 3.3.3. ADRs

**Figure 5:**
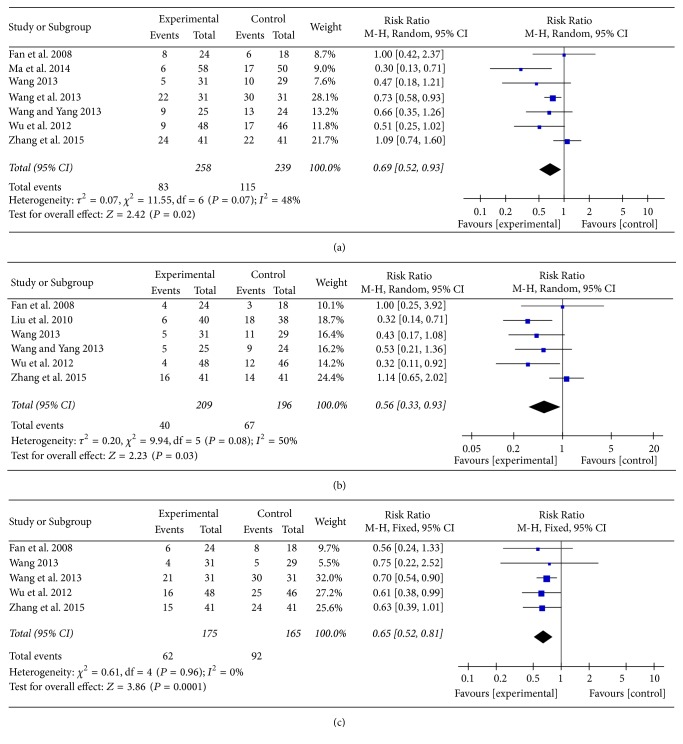
The forest plot of the three main ADRs: (a) nausea and vomiting; (b) diarrhea; (c) leukopenia.

There were 12 RCTs that referred to this outcome. The main ADRs were nausea and vomiting (7 RCTs) [14, 18–20, 22, 23, 25], diarrhea (6 RCTs) [17–20, 23, 25], and leukopenia (5 RCTs) [14, 18, 19, 23, 25]. Meta-analysis result manifested that there was a significant statistical difference between two groups. Compared with the control group, BJOEI group could be more effective in alleviating nausea and vomiting (Figure 5(a)), diarrhea (Figure 5(b)), and leukopenia (Figure 5(c)).

With regard to other ADRs such as myelosuppression, neutropenia, thrombopenia, and liver damage, BJOEI group was also superior compared with control group. However, no statistical significance was detected in terms of neurovirulence, renal damage, hand-foot syndrome, and oral mucositis. More detailed data for other ADRs was summarized in Table 2.

#### 3.3.4. Publication Bias

**Figure 6:**
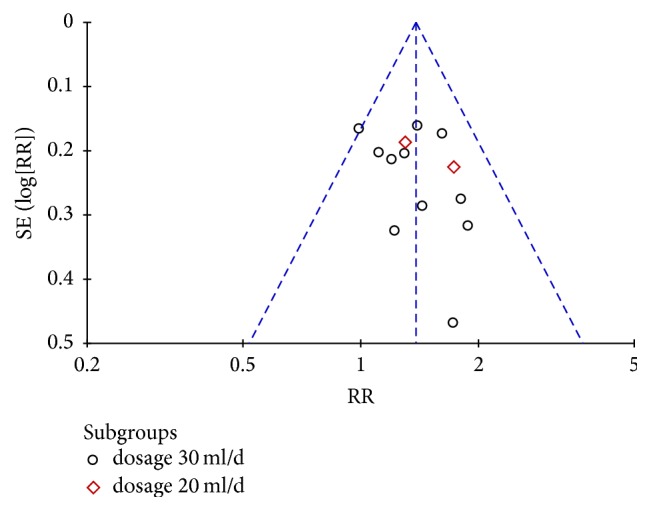
Funnel plot of the clinical total effective rate.

A funnel plot on publication bias for clinical total effective rate was displayed in Figure 6, and the result of Egger's test (*t* = 1.48, *P* = 0.168 >0.05) and Begg's test (*z* = 0.79, *P* = 0.428 >0.05) indicated no evidence of significant publication bias.

#### 3.3.5. Sensitivity Analysis

**Figure 7:**
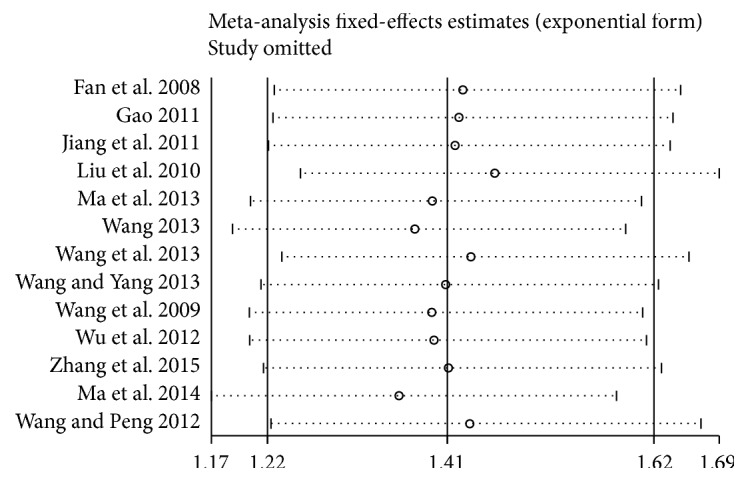
Sensitivity analysis in clinical total effective rate.

For clinical total effective rate, a sensitivity analysis was carried out to verify the stability of result, which was done by excluding RCTs seriatim at a time to resynthesize the data. As Figure 7 signified, sensitivity analyses revealed that no individual studies significantly affected the clinical total effective rate, which indicated statistically robust results.

## 4. Discussion

This meta-analysis unveiled that, on the basis of chemotherapy, the use of BJOEI can significantly improve clinical total effective rate and the performance status of GC patients. With regard to ADRs, BJOEI combined with chemotherapy owned the property to prominently relieving nausea and vomiting, diarrhea, neutropenia, neurotoxicity, and so on. However, there was no significant difference in the incidence of oral mucositis and hand-foot syndrome between BJOEI group and control group.

GC is one of the most common malignancies with high mortality in the world; surgical treatment is the therapeutic modality that offers the greatest possibility of cure for patients with GC; besides, chemotherapy and radiotherapy are important therapeutic options for patients who are suffering from distant metastases or unable to receive surgery [29, 30]. Nevertheless, some of the chemotherapy regimens with higher efficacy have more adverse reactions, and the prolongation of median survival time is not obvious [31]. Thus, how to reduce the burden of toxicity and achieve higher quality of life is the top priority on the clinical research agenda. As complementary and alternative medicine, TCM has become one of main methods for cancer comprehensive treatment. BJOEI was made of fatty oil extracted from* Brucea* fruit through petroleum ether, in which the oleic acid owned strong anticancer activity [32]. The anticancer activity of BJOEI might be attributed to the following properties: inducing apoptosis, disturbing the cell cycle, disrupting the cellular energy metabolism, and depressing the expression of vascular endothelial growth factor [7]. Consequently, it has the effect of killing and inhibiting cancer cells and can promote both humoral and cellular immunity but does not harm normal cells [33–35]. Zhang [36] examined the effect of* Brucea javanica* oil emulsion on HGC-27 cell proliferation of GC cells in vitro by MTT method; the results show that the* Brucea javanica* oil emulsion can significantly inhibit tumor cell proliferation and effectively inhibit the migration and invasion of human GC cell line HGC-27. At the same time, the drug resistance of human gastric adenocarcinoma with vincristine resistant cells is reversed [37]. Thus the conclusion of our study that BJOEI plus chemotherapy has the effect of enhancing efficacy and reducing ADRs was supported by pharmacological research and TCM therapeutic principal.

In the database, there are 2 systemic reviews focused on the efficacy of BJOEI for treating GC [38, 39], which contained 7 RCTs and 9 RCTs, respectively. The previous two researches have compared clinical total effective rate, improvement of performance status, and incidence of ADRs. By contrast, our research unveiled more detailed ADRs; these results testified that BJOEI combined with chemotherapy would be advantageous for relieving ADRs. Compared with Zhou Jiupeng's research [35], this meta-analysis gave clear indication of BJOEI's dosage. Beyond that, we also owned the following advantages: firstly, we updated the search date to January 9, 2017, and included a total of 13 RCTs by means of a relative comprehensive retrieval. Secondly, we formulated strict eligible criteria. All included patients were diagnosed with GC in specific criteria and evaluated the effectiveness in congruent standard and received the same interventions as well; it ensured the identical base line and boosted the validity of pooled data. Thirdly, subgroup analysis based on the diverse dosages revealed that there was favorable consistency between two subgroups, which indicated that, in comparison with chemotherapy alone, BJOEI intervention yielded a better result for improving clinical total effective rate regardless of dosages.

At the same time, the limitations to our study should be considered. Firstly, the 13 documents included are all Chinese literatures; only 9 RCTs referred to “random” for the grouping method and did not describe the specific random grouping method. All literatures had not referred to the use of blinding, and the quality of literatures is relatively general. Secondly, all of the studies which were included in the literature and published by the database had some publication bias. Although the large-sample and small sample randomized controlled trials were more evenly distributed, they also could affect the publication bias. Finally, due to the lack of sufficient information about patients' sex and age, we were unable to conduct subgroup analysis according to these included RCTs. Therefore, it is uncertain whether these discrepancies may influence the result to a certain extent and the results should be interpreted with caution.

## 5. Conclusion

To summarize, this study indicated that BJOEI combined with chemotherapy regimen seemed optimal for patients with GC in improving clinical total effective rate, performance status, and relieving ADRs. However, our findings should be confirmed by more prospectively designed, large-sample, and multicenter RCTs.

## Figures and Tables

**Table 1:** The basic characteristics of the included studies.

Study ID	Sex (M/F)	AVG age	*N* (E/C)	Therapy of experiment	Therapy of control	Course (d)	Outcomes
Jiang et al. 2011 [13]	41/23	32–64	32/32	BJOEI 30 ml + XELOX	XELOX	21 d	① ②
Wang et al. 2013 [14]	33/29	50.2	31/31	BJOEI 30 ml + PTX + ECX	PTX + ECX	21 d	① ②
Ma et al. 2013 [15]	38/12	60.0	25/25	BJOEI 30 ml + S1 + DDP	S1 + DDP	14–21 d	①
Wang et al. 2009 [16]	25/18	70–85	22/21	BJOEI 30 ml + UFT + BC	UFT + BC	30 d	① ②
Liu et al. 2010 [17]	56/22	29–71	40/38	BJOEI 30 ml + DX	DX	21 d	① ②
Wu et al. 2012 [18]	/	31–82	48/46	BJOEI 30 ml + FOLFOX4	FOLFOX4	14 d	① ②
Fan et al. 2008 [19]	27/15	70–85	24/18	BJOEI 30 ml + improved FOLFOX4	Modified FOLFOX4	21 d	①②③
Wang and Yang 2013 [20]	26/21	31–75	24/23	BJOEI 30 ml + FOLFOX4	FOLFOX4	14 d	① ② ③
Wang and Peng 2012 [21]	80/50	54.7	65/65	BJOEI 20 ml + OXA + CF + Tegafur	OXA + CF + Tegafur	21 d	① ②
Ma et al. 2014 [22]	88/20	46.8	58/50	BJOEI 20 ml + XELOX	XELOX	21 d	① ② ③
Wang 2013 [23]	40/20	52.0	31/29	BJOEI 30 ml + FOLFOX4	FOLFOX4	14–30 d	① ② ③
Gao 2011 [24]	29/23	32–79	26/26	BJOEI 30 ml + MC/CF	MC/CF	28 d	① ②
Zhang et al. 2015 [25]	54/28	68.7	41/41	BJOEI 30 ml + XELOX	XELOX	21 d	① ② ③

*Note*. M: male; F: female; E: experimental group; C: control group; CF: Leucovorin Calcium; BJOEI: *Brucea javanica* oil emulsion injection; PTX: Taxol; ECX: capecitabine; DDP: cisplatin; UFT: uracil-FT-207; BC: folic acid; ①: clinical total effective rate; ②: ADRs; ③: performance status.

**Table 2:** Meta-analysis results of other ADRs.

ADRs	*n*	Effect Model	RR [95% CI]	*z*	*P*
Myelosuppression	2	Fixed	0.49 [0.34, 0.69]	3.99	<0.0001
Neutropenia	2	Fixed	0.48 [0.27, 0.86]	2.46	0.01
Thrombopenia	4	Fixed	0.65 [0.46, 0.91]	2.54	*P* = 0.02
Liver damage	4	Fixed	0.50 [0.27, 0.92]	2.22	0.03
Neurovirulence	4	Random	0.72 [0.42, 1.21]	1.25	0.21
Renal damage	2	Fixed	0.54 [0.24, 1.22]	1.49	0.14
Hand-foot syndrome	4	Fixed	0.78 [0.57, 1.08]	1.48	0.14
Oral mucositis	2	Fixed	0.82 [0.34, 1.97]	0.44	0.66
